# Rebuilding a Sustainable Nursing Workforce: From Evidence to Action

**DOI:** 10.1097/jnr.0000000000000709

**Published:** 2025-09-26

**Authors:** Piao-Yi Chiou

The global nursing shortage remains a critical challenge for health systems, with a persistent deficit of 5.8 million nurses despite an expansion in the workforce from 27.9 million nurses in 2018 to 29.8 million in 2023 (World Health Organization, 2025). High global turnover and intention-to-leave rates (estimated at 15.2% and 38.4%, respectively; Mafula et al., 2025) as well as the disengagement of nearly 38% of licensed nurses in Taiwan (Taiwan Union of Nurses Association, 2025) further underscore the persistent and debilitating nature of this nursing shortage. Evidence indicates inadequate staffing not only intensifies the burden on existing teams but also elevates adverse event rates and compromises patient safety (Adamuz et al., 2025), reinforcing the urgency for integrated strategies that combine supportive work environments and professional development opportunities.

Ten studies in this issue address the intertwined priorities of sustaining the nursing workforce and advancing care quality. Drawing on both observational and interventional evidence, they highlight the importance of integrating supportive leadership, structured ethics communication and support, ongoing professional development, and technology-enabled well-being initiatives to strengthen engagement, reduce attrition, and reinforce professional identity. Collectively, these studies demonstrate how evidence-based approaches applied across diverse populations and care settings can refine diagnostic precision, improve functional and psychosocial outcomes, and foster family-centered care. Together, they provide a strategic framework for translating research into targeted, context-sensitive actions that empower nurses to deliver high-quality, holistic care while ensuring the long-term vitality of the nursing profession.
